# Translating the Game: Ribosomes as Active Players

**DOI:** 10.3389/fgene.2018.00533

**Published:** 2018-11-15

**Authors:** Piera Calamita, Guido Gatti, Annarita Miluzio, Alessandra Scagliola, Stefano Biffo

**Affiliations:** ^1^INGM, National Institute of Molecular Genetics, “Romeo ed Enrica Invernizzi”, Milan, Italy; ^2^Dipartimento di Bioscienze, Università Degli Studi Di Milano, Milan, Italy

**Keywords:** ribosomal proteins, ribosomopathies, ribosome heterogeneity, metabolism, Shwachman-diamond syndrome, eIF6, RACK1

## Abstract

Ribosomes have been long considered as executors of the translational program. The fact that ribosomes can control the translation of specific mRNAs or entire cellular programs is often neglected. Ribosomopathies, inherited diseases with mutations in ribosomal factors, show tissue specific defects and cancer predisposition. Studies of ribosomopathies have paved the way to the concept that ribosomes may control translation of specific mRNAs. Studies in *Drosophila* and mice support the existence of heterogeneous ribosomes that differentially translate mRNAs to coordinate cellular programs. Recent studies have now shown that ribosomal activity is not only a critical regulator of growth but also of metabolism. For instance, glycolysis and mitochondrial function have been found to be affected by ribosomal availability. Also, ATP levels drop in models of ribosomopathies. We discuss findings highlighting the relevance of ribosome heterogeneity in physiological and pathological conditions, as well as the possibility that in rate-limiting situations, ribosomes may favor some translational programs. We discuss the effects of ribosome heterogeneity on cellular metabolism, tumorigenesis and aging. We speculate a scenario in which ribosomes are not only executors of a metabolic program but act as modulators.

## Introduction

Translation the process by which mRNAs are translated into proteins by ribosomes. Eukaryotic ribosomes are evolutionarily conserved ribozymes constituted by ribosomal proteins (RPs) and rRNAs, whose structure has been spectacularly resolved (Ben-Shem et al., 2011; Klinge et al., 2011; Khatter et al., 2015). Ribosome biogenesis is a massive process occurring in the nucleolus of all cells. Recent progress, combining biochemical techniques, with structural and genetic evidence, has shown that ribosome synthesis is catalyzed and coordinated by more than 200 biogenesis factors. Ribosome biogenesis, therefore, proceeds through precise assembly steps that include several quality checkpoints, both in the nucleus and in the cytoplasm (Kressler et al., 2017; Pena et al., 2017). Furthermore, impairment of these checkpoints leads to defects in maturation that are associated with disease (Narla and Ebert, 2010; Ruggero and Shimamura, 2014).

In the cytoplasm, ribosomes are thought to constitute the hardware of the protein synthesis machinery, which fulfills its activity through four main phases: initiation, elongation, termination, and recycling. The initiation step is one of the most important steps of translation regulation, involving initiation factors, mRNAs, tRNAs, and ribosomes. Briefly, 40S subunits directly bind mRNAs in a way that is dependent on initiation factors and on mRNA structure and, after mRNA binding and scanning to an appropriate start codon, 60S subunits are recruited. Several studies elucidated how translation initiation is affected by alteration in mRNAs-binding factors (Loreni et al., 2014; Chu et al., 2016; Truitt and Ruggero, 2016) and by different features in mRNAs structures, i.e., Untranslated regions (UTRs). Recently, also tRNA has been linked to selective translation, and reprogramming of metabolism since codon reprogramming leads to HIF1α synthesis and an increase of glycolytic factors (Rapino et al., 2018).

Evidences that ribosomes exist in different forms in different cell types or during different stages of development (Milne et al., 1975; Bortoluzzi et al., 2001; Volarevic and Thomas, 2001) have suggested the presence of ribosome heterogeneity. It has been recently demonstrated that mutations in some RPs result in selective translation (Shi and Barna, 2015) and mutations in proteins causing an impairing in ribosome maturation and function, as in the case of ribosomopathies, show a specific mRNA translation signature (Brina et al., 2015; In et al., 2016) In conclusion, in recent years there has been growing evidence that translation is driven by ribosome heterogeneity, manifested as ribosome populations differing in ribosomal components. In this review we discuss ribosome heterogeneity in physiological, and pathological conditions, highlighting the role of translation machinery in driving the last step of the molecular biology central dogma, which elects ribosomes as players in specific mRNAs translation.

### Ribosome Heterogeneity in Physiological Conditions May Account for Differential Translation

This topic has been recently discussed (Genuth and Barna, 2018a,b) and we will give a simple summary of some perspectives. Ribosomes are constituted by approximately 80 RPs. For many years now, it is known that the relative abundance of different RPs, in different tissues, or in different growth conditions, may vary (Milne et al., 1975; Bortoluzzi et al., 2001; Volarevic and Thomas, 2001). This is a *sine qua non*-condition for ribosomal heterogeneity. An obvious alternative explanation for an imbalance of the stoichiometry of RPs within a cell is that RPs perform ribosome-independent functions. An experimental complexity is, therefore, to define whether a differential translation is due to the direct action of heterogeneous ribosomes or to regulatory pathways affected by free RPs. This is the case for RACK1 that was originally isolated as a PKC receptor (Ron et al., 1994; Gallo and Manfrini, 2015). RACK1 is a structural protein of 40S subunits (Gerbasi et al., 2004), involved in several extraribosomal functions (Mamidipudi et al., 2004; Robles et al., 2010; Wehner et al., 2011; Gandin et al., 2013; Fei et al., 2017). RACK1 may affect the efficiency of ribosomes directly (Ceci et al., 2003; Shor et al., 2003; Guo J. et al., 2011; Dobrikov et al., 2018a,b) or indirectly through signaling pathways (Gandin et al., 2013; Volta et al., 2013). In conclusion, data demonstrate that in physiological conditions, ribosomal networks may be more complex than expected and perform choices in translational regulation.

Ribosomal heterogeneity exists in physiological conditions. Accurate proteomics studies have identified sub-stoichiometric relationships within translating polysomes (Shi et al., 2017), showing that ribosomes may preferentially translate specific mRNAs. An experimental validation shows that ribosomes devoid of either RPS25 (eS25) or RpL10A (uL1), *in vivo*, translate specific mRNAs. Mechanistically, this study shows that the 60S subunits may affect mRNA recruitment through the binding of RPL10A (uL1) to IRES (Internal Ribosome Entry Site) sequences in the 5^′^UTR (Shi et al., 2017). In monocytes, interferon gamma driven phosphorylation results in RPL13A (uL13) detachment, but here it is still unknown whether ribosomes devoid of RPL13A (uL13) are able to translate selectively (Jia et al., 2012). Furthermore, RPL10 (uL16) R98S mutant leukemia cells are able to survive high oxidative stress levels by increasing IRES-dependent BCL-2 translation (Kampen et al., 2018).

Thus, the concept of a monolithic ribosome (Moore et al., 1968; Yusupova and Yusupov, 2017) may be accompanied by the existence of a more flexible ribosomal platform that performs further tuning on gene expression (Shi and Barna, 2015).

### Ribosome Heterogeneity in Pathological Conditions Affects Translation and Gene Expression

Ribosomopathies are inherited diseases caused by the loss of ribosomal component functionality. Some examples of ribosomopathies include Diamond-Blackfan Anemia syndrome (DBA), Shwachman-Diamond syndrome (SDS), Treacher Collins syndrome, 5q-myelodysplastic syndrome, and Dyskeratosis Congenita (DKC). Notably, all of these syndromes are characterized by variably penetrant phenotypes in which specific tissue deficits are found (Narla and Ebert, 2010). Early on, it was shown that DKC1 mutations reduce pseudouridylation and impair IRES mediated translation (Yoon et al., 2006).

As a case for study, we will focus our discussion on SDS. Signs of SDS include a peculiar exocrine pancreatic insufficiency, along with neutropenia and variable abnormalities in the skeleton and other organs. In addition, SDS is characterized by a reduction in growth, accompanied by an increased incidence of Acute Myeloid Leukemia, (AML; Dror, 2008). At the ribosomal level, SDS is characterized by the partial loss of free 60S ribosomal subunits due to, in most cases, mutations in the *SBDS* gene that is necessary for 60S maturation (Boocock et al., 2003; Wong et al., 2011). In a minority of cases, mutations of *EFL1p*, which acts in synergy with *SBDS*, have been found (Stepensky et al., 2017; Tan et al., 2018). Overall, the reduced functionality of 60S ribosomes is a common theme for SDS (Warren, 2018). All together these findings generate three questions: (a) how the loss of functionality of ubiquitous 60S ribosomes can generate tissue-specific defects, (b) how specific translational programs can be affected by the lack of 60S subunits, (c) how can we reconcile increased tumor with reduced growth.

Addressing this last question helps to put in the right context the other two. We have recently demonstrated in our lab that cells with mutant *Sbds* have reduced colony formation ability and are transformed less efficiently by oncogenes (Calamita et al., 2017). In this context, we demonstrated that *Sbds* deficiency directly acts by reducing the maximal oncogenic and translational capability of cells (Calamita et al., 2017). The paradox of reduced growth associated with tumor predisposition may not necessarily be associated with specific translation in tumor cells, but with a general impairment of tissue homeostasis that favors the appearance of mutant clones. For instance, increased tumor formation is observed in immunocompromised individuals (Verhoeven et al., 2018). To support this interpretation, the relationship between neutropenia and AML was described by different groups (Freedman et al., 2000; Link et al., 2007; Touw and Beekman, 2013). In conclusion, different cell types can be differentially affected by the reduction of RPs, i.e., thresholds can be different depending on the specific cellular demand of ribosomes for translation.

The question of the mechanism by which defects in 60S ribosomes lead to differential translation is more challenging since to our knowledge mRNA selection is driven by 40S subunits, prior to 60S engagement. However, the effects of 60S levels on specific translation are pervasive, and, as described before, IRES mRNA binding can be affected by RPL10 (uL16). In the case of *Sbds* depletion, characterized by reduced free 60S, two studies have addressed the question of preferential translation performing either microarray (Nihrane et al., 2009), or RNA-Seq on polysomes (Calamita et al., 2017). In addition, a reporter-based study has addressed the effect of *SBDS* depletion on reinitiation (In et al., 2016). Together, these studies support a model in which the SBDS deficiency reduces free 60S levels diminishing the maximal translational capability, and simultaneously changing translational selectivity. In this context, mRNAs that are intrinsically poorly translated because of uORFs (upstream Open Reading Frames) that require reinitiation are particularly disfavored. Similarly, mouse models have underscored that the reduction of 60S RPs affects the translational program of IRES containing mRNAs (Barna et al., 2008; Kondrashov et al., 2011; Xue et al., 2015).

Finally, mathematical modeling of translation suggests that a quantitative reduction in the translational output may result in strong alterations of specific mRNA translation due to stochastic events (Heinrich and Rapoport, 1980; Mills and Green, 2017). We conclude that some mRNAs can be particularly sensitive to ribosomal availability, and we speculate that this property has been evolutionarily exploited to connect ribosomes with other cellular events. What we still lack is understanding the precise mechanisms.

### A Common Theme for the Regulatory Function of Ribosomes?

Metabolic pathways are necessary for converting essential nutrients into energy and macromolecules that sustain cell growth and proliferation. Nutrients and metabolic pathways control all facets of cellular functions. Nutrient and growth factors converge on the translational machinery through signaling pathways that, in turn, regulate the synthesis of ribosomes and the activity of translation factors (Roux and Topisirovic, 2018). Then, translation factors crosstalk to metabolic choices (Biffo et al., 2018). Some well-established observations are the following. mTORC1 controls mitochondrial activity and biogenesis by selectively promoting translation of nucleus-encoded mitochondria-related mRNAs, via inhibition of the eukaryotic translation initiation factor 4E (eIF4E)-binding proteins (4E-BPs; Morita et al., 2013). ROS generation is also controlled partly at the translational level through eIF4E (Truitt et al., 2015). Glutamine metabolism is controlled by eIF4B-mediated translation downstream of mTORC1 pathway (Csibi et al., 2014). eIF3 complex mediates energy metabolism (Shah et al., 2016). Rate-limiting initiation factors that link 60S ribosome biogenesis to translation as eIF6 hierarchically control lipid synthesis and metabolism, through uORF and G/C rich 5^′^UTR sequences (Brina et al., 2015). eIF5A2 accelerates lipogenesis in hepatocellular carcinoma (Cao et al., 2017). In general, translation and metabolism are dysregulated in a coordinated fashion (Leibovitch and Topisirovic, 2018), and initiation factors may act upstream of metabolic reprogramming (Biffo et al., 2018). The next question is whether ribosomes also control metabolic pathways.

In Zebrafish, *rpl11* mutation decreased the glycolytic rate and the lower activity of glycolytic enzymes is rescued by p53 inhibition (Danilova et al., 2011). Moreover, defects, mutations or imbalance of RPs stabilized p53 and changed metabolic flux, specifically by decreasing glycolysis and enhancing aerobic respiration (Deisenroth and Zhang, 2011). Albeit these data do not support a direct crosstalk between ribosome activity and metabolism, they suggest overall that when the translation machinery is perturbed, coordinated pathways involved in cell homeostasis and metabolism are also altered.

**FIGURE 1:**
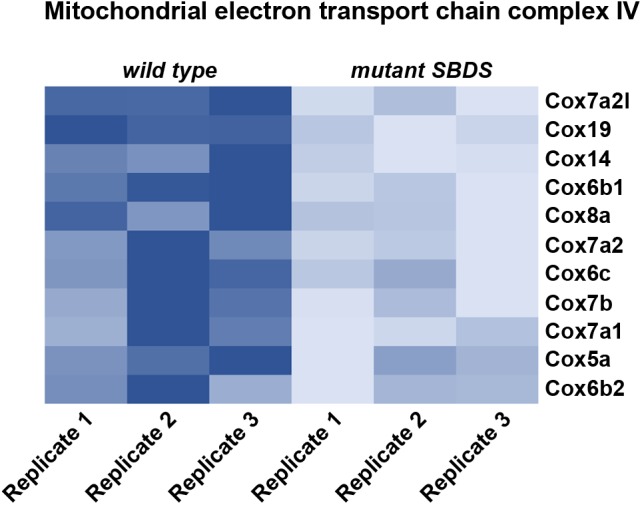
Heat map representing relative gene expression levels in a cellular model for Shwachman Diamond Syndrome. We re-infected cells bearing the mutation R126T/R126T (corresponding to one of the most common mutations associated with Shwachman Diamond Syndrome) in the *Sbds* gene (*Sbds^R126T/R126T^* MEFs) with either wild type *Sbds* (*Sbds^RESCUE^*), or mock control (*Sbds^MOCK^*). Heat map represents relative gene expression levels of genes associated with mitochondrial electron transport chain complex IV, showing an overall reduction in *mutant Sbds^MOCK^* cells, indicating an impairment in ATP production. Heatmap is based on RNASeq raw data available at www.ebi.ac.uk/arrayexpress with accession number ID E-MTAB-5089, and analyzed in our previous work (Calamita et al., 2017).

Recently, it has been shown that SDS cells display an impairment in Complex IV activity, which causes an oxidative phosphorylation metabolic defect, with a consequent decrease in ATP production (Ravera et al., 2016). The authors suggest an indirect effect of *SBDS* mutation on energy production levels, indicating a possible role of calcium homeostasis in altering complex IV activity. In our lab we performed a characterization of a cellular model for SDS by immortalizing Mouse Embryonic Fibroblasts (MEFs; Calamita et al., 2017) derived from an SDS mouse model carrying the R126T mutation in homozygosity (*Sbds^R126T/R126T^* MEFs) (Tourlakis et al., 2012). Briefly, we established a model for studying SBDS function by retrasducing *Sbds^R126T/R126T^* MEFs with either wild-type *Sbds* (*Sbds^RESCUE^*), or mock control (*Sbds^MOCK^*) vectors. In this way, we can separate direct events due to a lack of SBDS from indirect effects. We confirmed a decrease in ATP levels associated with *Sbds* mutation. In addition, our RNA-Seq analysis revealed that genes belonging to complex IV were less expressed when *Sbds* was mutated (Figure 1). This downregulation could explain an impairment in cytochrome C oxidase activity and a consequent defect in ATP production. Moreover, there is a defect in oxygen consumption rate in SDS cells (Ravera et al., 2016; Calamita et al., 2017), as well as a reduction in the lactate/pyruvate ratio (Calamita et al., 2017). The mechanistic connection between ribosome function and the metabolic effects of its impairment is still to be clarified. Overall, a reduction in ribosomal efficiency seems to associate with a reduction in energy levels and lipid biosynthesis. We suggest that ribosomal capability has coevolved with other cellular functions and, specifically, ribosomes are intimately linked to nutrient levels and cellular growth.

The connection between ribosomes and growth is indeed strong and well-known. In *Drosophila melanogaster* the haploinsufficiency of RPs results in the *minute* phenotype, which includes short and thin bristles and smaller flies (Lambertsson, 1998; Marygold et al., 2007). Moreover, as shown by a myriad of papers, depletion of RPs causes a delay/arrest in cell cycle progression. In several cases, the regulation of growth is associated with ribosome independent function of RPs (Dai and Lu, 2004; Mamidipudi et al., 2004, Dutt et al., 2011; Yao et al., 2016). In other cases, the inhibition of growth has been directly linked to translational control driven by ribosomes (Barna et al., 2008). Depletion of different RPs may result in different types of inhibition of cell cycle progression, in line with the concept of heterogeneity in ribosomes (Badhai et al., 2009). Conversely, nucleolar enlargement grossly equals an increased production of ribosomes and is observed in many cancers (Montanaro et al., 2008). In many models, some heterozygous deletions of RPs reduce tumor growth (Barna et al., 2008; Chen et al., 2014; Wilson-Edell et al., 2014), while some others are associated with cancer development as demonstrated for the first time in zebrafish mutants for RPs in 2004 (Amsterdam et al., 2004). In the last years, several somatic mutations have been linked to tumor progression and belong to both 60S subunits such as RPL5 (uL18) and RPL10 (uL16) (De Keersmaecker et al., 2013), RPL 11 (uL5) (Tzoneva et al., 2013; Fancello et al., 2017), RPL22 (eL22) (Rao et al., 2012) and RPL 23 (uL23) (Fancello et al., 2017) and to 40S subunits such as RPS15 (uS19) (Landau et al., 2015; Ljungstrom et al., 2016), RPS27 (eS27) (Dutton-Regester et al., 2014) and RPSA (uS2) (Fancello et al., 2017). On the contrary, RPs overexpression has been also identified in cancer progression (Artero-Castro et al., 2011; Guo X. et al., 2011; Yang et al., 2016). Several recent reviews provide a comprehensive discussion on how, in some cases, loss of RPs contributes to cancer (Sulima et al., 2017; Genuth and Barna, 2018a; Pelletier et al., 2018).

The ribosomal apparatus also appears to affect longevity. Alterations in ribosomal protein expression result in an extension of eukaryotic lifespan (Hansen et al., 2007; Steffen et al., 2008).

In short, the persistent link between ribosomal function in growth and metabolism makes us speculate that there may be a yet-to-be-unveiled mechanistic connection. We favor a model in which mRNAs important for cell cycle progression or for key metabolic pathways contain UTRs that have coevolved with the translational machinery in order to be preferentially translated in conditions of optimal ribosomal capability. In this context, ribosomal heterogeneity may further tune the cell^′^s translational capabilities.

### Mitochondrial Ribosomes

Several mitochondrial ribosome proteins are also involved in different cellular processes, such as cell cycle, apoptosis and mitochondrial homeostasis regulation. Mutations in mt-RPs genes are associated with mitochondrial dysfunctions and disorders (Saada et al., 2007; Smits et al., 2011; Serre et al., 2013; Menezes et al., 2015; Richman et al., 2015). For instance, mutant *MRPS16 (bS16m)* causes mitochondrial respiratory chain disorders (Miller et al., 2004) and loss of *MRPL10 (uL10m)* diminished mitochondrial respiration and intracellular ATP levels (Li et al., 2016). In addition, a recent study claims the regulation of cytoplasmic protein homeostasis by mitochondrial translation (Suhm et al., 2018). These studies elucidate the fact that a crosstalk between the cytoplasmic and the mitochondrial ribosomal machinery may be present.

## Conclusion

**FIGURE 2:**
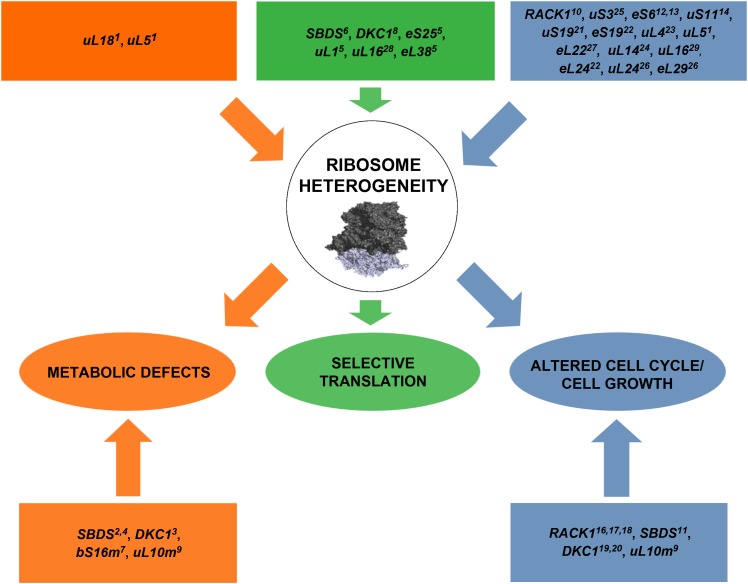
A schematic model representing the list of genes whose mutations perturb ribosome machinery. The color indicates the associated phenotype, specified in ovals in the third row. In some cases, the effect is associated or supposed to be associated with extra-ribosomal functions of mutated genes (listed in squares in the fourth row). Briefly, alterations in ribosome biogenesis and/or mutations in ribosomal proteins are responsible for metabolic changes, abnormal cell cycle progression/cell growth, and selective translation. Ribosomal subunits adapted from 40S (Lomakin and Steitz, 2013) PDB code 5ANB to 60S (Weis et al., 2015) PDB code 4KZX. Orange color indicates the flux of alterations converging to metabolic defects, green color the flux converging to selective translation and blue color the one converging to altered cell cycle/cell growth. The exploration of effects of RP lesions on cell cycle, translation, and cell metabolism is a highly active area of research and novel effects of RP lesions still need to be discovered. ^1^Danilova et al., 2011; ^2^Calamita et al., 2017; ^3^Angrisani et al., 2018; ^4^Ravera et al., 2016; ^5^Shi et al., 2017; ^6^In et al., 2016; ^7^Miller et al., 2004; ^8^Yoon et al., 2011; ^9^Li et al., 2016; ^10^Ceci et al., 2012; ^11^Menne et al., 2007; ^12^Volarevic et al., 2000; ^13^Sulic et al., 2005; ^14^Dutt et al., 2011; ^15^Teng et al., 2013; ^16^Hermanto et al., 2002; ^17^Mamidipudi et al., 2004; ^18^Fei et al., 2017; ^19^Alawi and Lin, 2013; ^20^Ge et al., 2010; ^21^Yao et al., 2016; ^22^Badhai et al., 2009; ^23^He et al., 2016; ^24^Dai et al., 2004; ^25^Yoon et al., 2006; ^26^Li et al., 2012; ^27^Stadanlick et al., 2011; ^28^Kampen et al., 2018; ^29^De Keersmaecker et al., 2013.

Ribosomes have been long considered as monolithic structures ensuring mRNAs translation in a passive way. Nowadays, it has been well established that ribosomes can affect not only mRNA selection but also other fundamental processes such as cell growth and lately, cell homeostasis and metabolism (Figure 2). There is an increasing number of studies evidencing that the inter-correlation between ribosomes and metabolic pathways leads to a common cellular phenotype. Since ribosomes are a rate-limiting component of the translational program, further studies are needed to elucidate specific molecular mechanisms by which ribosome heterogeneity, supported by the translational apparatus, sustain cell growth and metabolic homeostasis.

## Author Contributions

SB and PC reviewed and edited the manuscript. SB, PC, GG, and AS reviewed the literature. SB, PC, and AM wrote the manuscript. GG conceived and prepared figures, and edited the manuscript. All authors contributed, read, and approved the manuscript.

## Conflict of Interest Statement

The authors declare that the research was conducted in the absence of any commercial or financial relationships that could be construed as a potential conflict of interest.
